# Naoxintong capsule for treating cardiovascular and cerebrovascular diseases: from bench to bedside

**DOI:** 10.3389/fphar.2024.1402763

**Published:** 2024-06-27

**Authors:** Wei-jian Zhang, Rui-qi Chen, Xuan Tang, Pei-bo Li, Jian Wang, Hai-ke Wu, Ning Xu, Ming-fei Zou, Sen-rong Luo, Zi-qi Ouyang, Zhi-kai Chen, Xu-xing Liao, Hao Wu

**Affiliations:** ^1^ Department of Neurosurgery, First People’s Hospital of Foshan, Foshan, Guangdong, China; ^2^ Guangdong Engineering and Technology Research Center for Quality and Efficacy Reevaluation of Post-market Traditional Chinese Medicine, Guangdong Key Laboratory of Plant Resources, School of Life Sciences, Sun Yat-sen University, Guangzhou, China; ^3^ Department of Neurosurgery, Foshan Sanshui District People’s Hospital, Foshan, Guangdong, China; ^4^ Department of Neurology, Foshan Hospital of Traditional Chinese Medicine, Foshan, Guangdong, China; ^5^ Second People’s Hospital of Foshan, Foshan, Guangdong, China; ^6^ Guangdong Medical University, Zhanjiang, Guangdong, China

**Keywords:** traditional Chinese medicine (TCM), naoxintong capsule (NXT), cardiovascular and cerebrovascular diseases (CCVD), chemical compositions, pharmacological properties, clinical applications

## Abstract

Naoxintong Capsule (NXT), a renowned traditional Chinese medicine (TCM) formulation, has been broadly applied in China for more than 30 years. Over decades, accumulating evidences have proven satisfactory efficacy and safety of NXT in treating cardiovascular and cerebrovascular diseases (CCVD). Studies have been conducted unceasingly, while this growing latest knowledge of NXT has not yet been interpreted properly and summarized comprehensively. Hence, we systematically review the advancements in NXT research, from its chemical constituents, quality control, pharmacokinetics, to its profound pharmacological activities as well as its clinical applications in CCVD. Moreover, we further propose specific challenges for its future perspectives: 1) to precisely clarify bioactivities of single compound in complicated mixtures; 2) to evaluate the pharmacokinetic behaviors of NXT feature components in clinical studies, especially drug-drug interactions in CCVD patients; 3) to explore and validate its multi-target mechanisms by integrating multi-omics technologies; 4) to re-evaluate the safety and efficacy of NXT by carrying out large-scale, multicenter randomized controlled trials. In brief, this review aims to straighten out a paradigm for TCM modernization, which help to contribute NXT as a piece of Chinese Wisdom into the advanced intervention strategy for CCVD therapy.

## Graphical Abstract

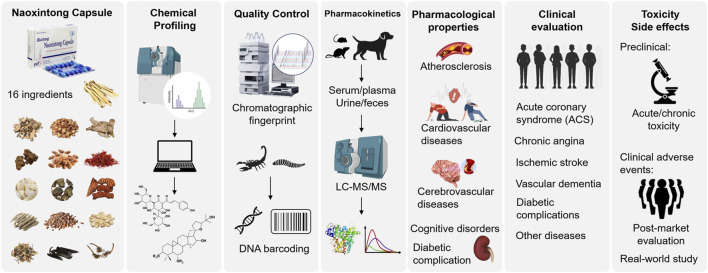

## 1 Introduction

Cardiovascular and cerebrovascular diseases (CCVD) are a large category of diseases that pose a great threat to public health, causing more than 20 million deaths worldwide each year (Hasani et al., 2023). However, the quality of survival management of CCVD is constantly low because CCVD patients often undergo an intricate, multifactorial pathogenesis, leading to high mortality and high degree of disability (Joseph et al., 2017). Nowadays, the conventional strategies for CCVD management mainly include platelet aggregation inhibitors to prevent thrombosis (e.g., aspirin and clopidogrel), hypolipidemic agents to sustain lipids homeostasis (e.g., statins or fibrates), and antihypertensive therapies to maintain blood pressure (e.g., β-blockers and ACE-I/angiotensin receptor blockers) as well. Many patients also need to take drugs such as metformin to control their blood glucose at the same time. Differences in drug use may be one of the main reasons for the large differences in morbidity and mortality of acute myocardial infarction or stroke in different income countries (Leong et al., 2017). However, many patients with CCVD require long-term or even lifelong medication after diagnosis. These kinds of patients often accompanied by a variety of underlying diseases, having various potential health risks under the current drug treatment strategies. Moreover, the complex disease complications also bring a huge healthcare burden to many patients’ families. Therefore, it is necessary to explore new safe and effective treatment options.

Traditional Chinese Medicine (TCM) draws an increasing interest worldwide for its complementary and alternative therapeutic effects compared with Western medicine (WM) and its remarkable ability to address various difficult diseases (Liu et al., 2015; Pan et al., 2022). TCM has been used as an effective intervention for thousands of years in the treatment of CCVD in Asia, accumulating a quantity of valuable medication experience and prescriptions, with at least more than 20 herbs commonly used in TCM reported to have strong cardiovascular and cerebrovascular protective effects (Hao et al., 2017; Zhang et al., 2022). Naoxintong capsule (NXT) is a modern TCM prescription derived from Buyang Huanwu Decoction, approved by the China Food and Drug Administration (CFDA) and included in the Chinese Pharmacopeia (2020 edition). NXT has been used for the treatment of CCVD for about 30 years. Accumulating evidence from animal experiments and clinical applications has confirmed the significant efficacy and safety of NXT (Han et al., 2019). As the representative prescription of co-treatment of CCVD, in addition to the remarkable efficacy and safety, NXT possesses distinct therapeutic advantages compared to traditional strategies for treating CCVD. For instance, compared with the conventional drugs, patients have a good tolerance and little resistance to NXT treatment (Han et al., 2019). Besides, NXT in combination with the conventional interventions helps to improve efficacy while reduce side effects (Yu et al., 2024). Consequently, NXT has achieved excellent efficacy feedback in the Chinese market over the past 30 years, with an annual sales volume of 1 billion yuan, making it a fairly well-known modern application of TCM in the treatment of CCVD. Over the years, TCM, represented by NXT, has accumulated a lot of valuable experience in the clinical treatment of CCVD, but the intrinsic reasons for the efficacy of TCM still need to be studied more deeply and thoroughly.

Post-marketing re-evaluation is an essential part of TCM modernization research, which re-articulates the accumulated experience of TCM with the theories of modern medicine. Re-evaluation of the quality and efficacy of TCM prescriptions after marketing is helpful to continuously improve the pharmaceutical process, interpret the pharmacological mechanism, and ensure the safety of drug use. NXT, as one of the most widely used TCMs with the abundant research base and the vast majority of evidence of efficacy in China, has accumulated a large number of results in modernization research, but it is undeniable that it still lacks some critical studies. In this review, we systematically sort out the current reports of NXT in terms of composition, quality control, pharmacokinetics, pharmacological activity, clinical application, etc. We aim to explore the research paradigm of TCM post-market re-evaluation by taking NXT as an example, as well as to provide ideas and directions for TCM modernization research and provide valuable references for new interventions or therapeutic ideas for CCVD.

## 2 Chemical profiles

NXT is a fine powder mixture containing 11 botanical drugs [*Astragalus membranaceus* (Fisch.) Bge. [Fabaceae, astragali radix], *Paeonia lactiflora* Pall. [Paeoniaceae, paeoniae radix rubra], *Salvia miltiorrhiza* Bunge [Lamiaceae, salviae miltiorrhizae radix et rhizoma], *Angelica sinensis* (Oliv.) Diels [Apiaceae, angelicae sinensis radix], *Conioselinum anthriscoides* ‘Chuanxiong’ [Apiaceae, chuanxiong rhizoma], *Prunus persica* (L.) Batsch [Rosaceae, persicae semen], *Carthamus tinctorius* L. [Asteraceae, carthami flos], *Spatholobus suberectus* Dunn [Fabaceae, spatholobi caulis], *Achyranthes bidentata* Blume [Amaranthaceae, achyranthis bidentatae radix], *Neolitsea cassia* (L.) Kosterm. [Lauraceae, Cinnamomi ranulus], and *Morus alba* L. [Moraceae, ramulus mori]], 2 kinds of resin drugs [*Boswellia sacra* Flück. [Burseraceae, olibanum], *Commiphora myrrha* (T. Nees) Engl. [Burseraceae, Myrrha]] and 3 kinds of zoological drugs [*Pheretima aspergillum* (E.Perrier). [Megascolecidae, pheretima], *Hirudo nipponica* Whitman [Hirudinidae, hirudo], and *Buthus martensii* Karsch [Buthidae, scorpio]]. The preparation method of NXT includes crushing the above 16 medicinal materials into fine powder, screening through mesh size of 80, then mixing in a certain proportion (details in Table 1), and finally putting into the capsule (Chinese Pharmacopoeia Commission, 2020). NXT is a prescription that fully complies with the principles of TCM formulas.

**TABLE 1:** The ingredients of NXT.

Chinese name	The role of herbs in formula (according to TCM theory)	Pharmaceutical name	Latin binomial	Content (mg/capsule)
Huangqi	Jun (monarch herb for tonifying qi)	Astragali Radix	*Astragalus membranaceus* (Fisch.) Bge.	66
Dilong	Chen (minister herb for clearing and activating the channels and collaterals)	Pheretima	*Pheretima aspergillum* (E.Perrier)	27
Shuizhi		Hirudo	*Hirudo nipponica* Whitman	27
Quanxie		Scorpio	*Buthus martensii* Karsch	13
Danggui	Zuo (adjuvant herb for activating blood circulation and removing blood stasis)	Angelicae Sinensis Radix	*Angelica sinensis* (Oliv.) Diels	27
Chuanxiong		Chuanxiong Rhizoma	*Ligusticum chuanxiong* Hort	27
Danshen		Salviae Miltiorrhizae Radix et Rhizome	*Salvia miltiorrhiza* Bunge	27
Chishao		Paeoniae Radix Rubra	*Paeonia veitchii* Lynch	27
Taoren		Persicae Semen	*Prunus persica* (L.) Batsch	27
Honghua		Carthami Flos	*Carthamus tinctorius* Linn	13
Ruxiang		Olibanum (Boswellia Carterii Resina)	*Boswellia carterii* Birdw	13
Moyao		Myrrha (Commiphora Resina)	*Commiphora myrrha* (Nees) Engl	13
Sangzhi	Shi (courier herb for guiding others herbs to the specific meridians)	Ramulus Mori	*Morus alba* L	27
Niuxi		Achyranthis Bidentatae Radix	*Achyranthes bidentata* Blume	27
Jixueteng		Spatholobi Caulis	*Spatholobus suberectus* Dunn	20
Guizhi		Cinnamomi Ranulus	*Cinnamomum cassia* Presl	20

The content of 16 Chinese traditional medical herbs of NXT, came from Chinese Pharmacopoeia 2020.

**FIGURE 1:**
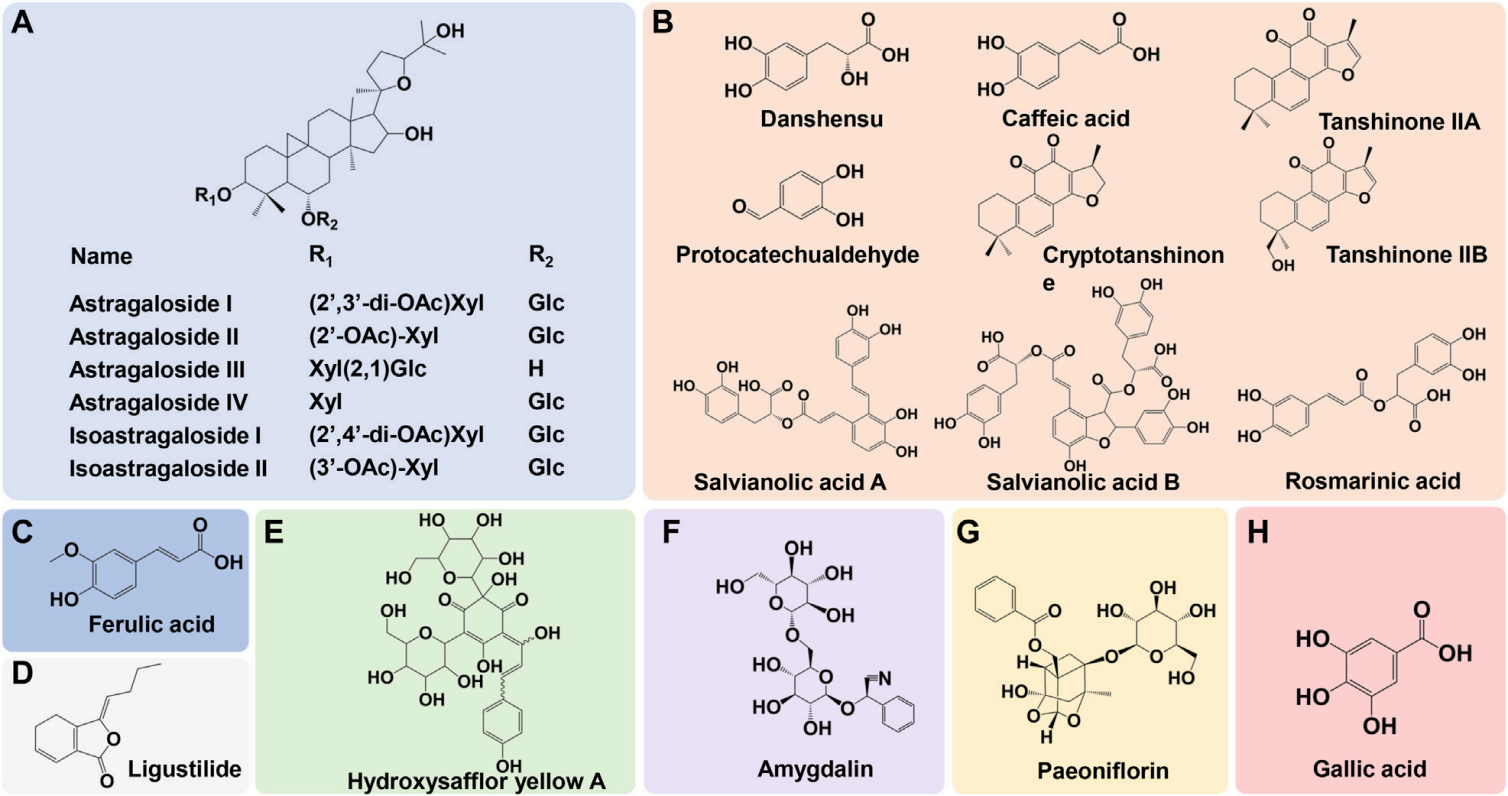
The primary constituents in NXT and corresponding source. **(A)** Astragali Radix, **(B)** Salviae Miltiorrhizar Radix et Rhizoma, **(C)** Angelicae Sinensis Radix/Chuanxiong Rhizoma, **(D)** Chuanxiong Rhizoma, **(E)** Carthami Flos, **(F)** Persicae Semen, **(G)** Paeoniae Radix Rubra, **(H)** Spatholobi Caulis/Paeoniae Radix Rubra.

Astragali Radix (Huangqi in Chinese) plays a key role in the formula of NXT. Astragali Radix mainly contains polysaccharides, flavonoids and saponins. The primary active components in Astragali Radix are astragalosides and iso-astragalosides, such as astragaloside I, II, III, IV, and iso-astragaloside I, II, which account for more than 80% of the total saponins (Zhang et al., 2021). For Astragali Radix, Calycosin-7-O-β-D-glucoside is the component commonly used for the chemical marker in the quality analysis, while astragaloside IV is assigned as a principal quality control index (Ping et al., 2017). The main chemical compositions of Pheretima (Dilong) include protein, amino acid, enzymes, lipids, trace elements and nucleotides. Lumbrukinase is one of the characteristic components of Pheretima, which possess the anticoagulant and thrombolytic effects (Xu et al., 2022). Hirudo (Shuizhi) mainly contains protein polypeptides, pteridines, glycolipids, carboxylic esters and free amino acids. Hirudin is the most active components in Hirudo, which is the strongest natural thrombin specific inhibitor so far (Shi et al., 2023). The main components of Scorpio (Quanxie) include buthotoxin, sterol derivatives, alkaloids, amino acids, lipids and terpenoid (Kyungjin et al., 2018). The primary active constituents of Salviae Miltiorrhizae Radix et Rhizoma (Danshen) are water-soluble phenolic acids and lipid-soluble diterpenoids. Contained phenolic acids mainly include danshensu, protocatechualdehyde, salvianolic acid A, B, C, D, E, F, G, as well as caffeic acid, rosmarinic acid, lithospermic acid, while diterpenoids are mainly tanshinone I, tanshinone IIA, tanshinone IIB, and cryptotanshinone (Su et al., 2015). The primary components in Angelicae Sinensis Radix (Danggui) are phthalides, phenolic acids and their derivatives, polysaccharides, phenylpropanoids and flavonoids. Among them, phenolic acids represented by ferulic acid and their derivatives are the main bioactive components, which are closely related to the pharmacological effect of Angelicae Sinensis Radix (Yi et al., 2009). The main chemical components of Chuanxiong Rhizoma (Chuanxiong) include volatile oils, phenolic acids, alkaloids, and polysaccharides. Ferulic acid, coniferyl ferulate, Z-ligustilide, senkyunolide A, I, H, as well as levistolide A are supposed to be the quality marker of Chuanxiong Rhizoma (Li et al., 2012). Paeoniae Radix Rubra (Chishao) mainly contains terpenoids, tannins, flavonoids and polysaccharides. Paeoniflorin serves as a primary quality control indicator of Paeoniae Radix Rubra (Yue et al., 2022). Persicae Semen (Taoren) mainly contains volatile oils, cyanogenic glycosides, flavonoids, sterols and saponins. Amygdalin is assigned as a principal quality control index of Persicae Semen (Yang et al., 2014). Carthami Flos (Honghua) mainly contains flavonoids, alkaloids, lignans, sterols and polysaccharides and hydroxysafflor yellow A is a chemical marker for its quality control (Qiao et al., 2021). The primary components in Olibanum (Ruxiang) and Myrrha (Moyao) are terpenoids and volatile oils (Xu et al., 2011; Feng et al., 2021). The main active components in Spatholobi Caulis (Jixueteng) are flavonoids, phenolic acids, phenylpropanoids, terpenoids and sterols (Guan et al., 2013). The main active components in Ramulus Mori (Sangzhi) are alkaloids and polysaccharides (Yang et al., 2015). Cinnamomi Ranulus (Guizhi) mainly contains phenylpropanoids, terpenoids, flavonoids and volatile oils (Liu et al., 2020). The primary active components in Achyranthis Bidentatae Radix (Niuxi) are polysaccharides and saponins. β-ecdysterone is assigned as a principal index of quality control of Achyranthis bidentatae (He et al., 2017). A brief illustration of the primary constituents in NXT were shown in Figure 1.

As a mixed mixture, NXT’s chemical composition is extremely complex. With the development of analytical techniques, researchers tried to clarify the chemical profiles of NXT via instruments with high throughput and high resolution in recent years. Using the ultra-high-pressure liquid chromatography coupled with linear ion trap-Orbitrap tandem mass spectrometry (UHPLC-LTQ-Orbitrap), Wang et al. identified the composition of NXT. As a result, a total of 178 compounds were detected, including 21 flavones, 6 flavone glycosides, 18 phenanthraquinones, and 22 terpenoids (Wang et al., 2015), while Ma et al. identified 81 compounds with the method of ultra-performance liquid chromatography/quadrupole time-of-flight mass spectrometry (UPLC/Q-TOF-MS) (Ma et al., 2016). The identified components of both two methods mainly included organic acids, amino acids, flavonoids, lactones and terpenoids. In terms of composition quantification, a method of Matrix Solid-Phase Dispersion (MSPD) combined with high performance liquid chromatography (HPLC) was established to simultaneously determine sixteen compounds. These compounds included phenolic acids (gallic acid, lithospermic acid, chlorogenic acid, ferulic acid, rosmarinic acid, 1,5-dicaffeoylqunic acid, salvianolic acid B, and 3,5-dicaffeoylqunic acid), flavonoids (formononetin, calycosin, and kaempferol-3-O-rutinoside), lactones (butyllidephthalide and ligustilide), monoterpenoids (paeoniflorin), phenanthraquinones (cryptotanshinone), and furans (5-hydroxymethylfurfural), which could be considered as potential chemical markers for quality control of NXT (Wang et al., 2018a). Recently, six active compounds including paeoniflorin, lithospermic acid, salvianolic acid B, Z-ligustilid, 3,5-dicaffeoylquinic acid, rosmarinic acid, were found to be accounted for a large proportion of thrombin/FXa inhibitory activity of NXT, which supposed to be anticoagulant quality markers in NXT (Du et al., 2022).

## 3 Quality control

Quality control is an indispensable part to ensure the safety and effectiveness of TCM. Currently, a series of strategies have been used to establish an appropriate quality control system, including qualitative identification, content determination, finger-prints, etc. Multiple technologies such as thin layer chromatography (TLC), high performance liquid chromatography (HPLC), gas chromatography (GC), and mass spectrometry (MS) have been widely applied to the study on quality standard of TCM.

### 3.1 Qualitative analysis

For NXT, in the Chinese Pharmacopoeia of 2020 edition, microscope is used for the identification of Carthami Flos, Angelicae Sinensis Radix, Astragali Radix, Cinnamomi Ranulus, Paeoniae Radix Rubra, Scorpio, and Spatholobi Caulis, while TLC is used to qualitatively identify Salviae Miltiorrhizar Radix et Rhizoma, Astragali Radix, Angelicae Sinensis Radix, Chuanxiong Rhizoma, Achyranthis Bidentatae Radix, Boswellia Carterii Resina, Spatholobi Caulis, and Cinnamomi Ramulus.

Chromatographic fingerprinting is an effective strategy to evaluate the intact quality of TCM medicinal materials and formula, which can reflect the complex chemical information contained in TCM more comprehensively. Using HPLC coupled with DAD detector, Su et al. established fingerprint-based quality control method for NXT with 33 common characteristic peaks, 13 of which were identified as mulberroside A, ligustrazine, catechin, paeoniflorin, calycosin-7-glucoside, hydroxysafflor yellow A, ferulic acid, rosmarinic acid, calycosin, salvianolic acid B, formononetin, tanshinone Ⅰ, tanshinone Ⅳ(Su et al., 2019), which were derived from twelve plant herbs in NXT, and could provide an effective method for quality control. However, due to the huge difference in components between plant herbs and zoological drugs, the sample preparation methods and the detection conditions are totally different, no components of zoological drugs were detected in this HPLC fingerprint. Indeed, it is extremely difficult for researchers to simultaneously determine the compounds belonging to sixteen medicinal materials in the formula.

Quality control of animal derived ingredients is also an essential approach to ensure the safety and effectiveness of NXT. DNA barcoding technology is a newly developed biological species identification technology in recent years. This technology uses a relatively short standard DNA sequence in the genome to identify species. It has been widely applied in the identification of Chinese medicinal materials from animals and plants. Most of the identified ingredients of NXT in the above studies are derived from the plant herbs. There has been a lack of reports on the identification of three zoological drugs (Hirudo, Scorpio, and Pheretima) in NXT and their protein components. In order to fill this gap, our group used DNA barcoding molecular identification technology to design the specific primers of Hirudo, Scorpio, and Pheretima, which can be used to identify whether the sample of NXT contains the corresponding authentic animal drugs, greatly improving the quality control of NXT. Using DNA barcoding technology, we developed a method to authenticate zoological drugs including Hirudo, Scorpio, and Pheretima in NXT (Zhu et al., 2019a; b; 2020). Specific primers were designed through mitochondrial cytochrome c oxidase subunit I (COI) gene sequence for genuine species, namely, Metaphire and Amynthas generas of Pheretima, *Whitmania pigra* and *Hirudo nipponica* of Hirudo, *Mesobuthus martensii* of Scorpio, respectively. Using specific primers, PCR amplification was performed for the genuine species and the common asulterants of Pheretima, Hirudo and Scorpio, as well as NXT. The obtained amplification sequences were sequenced and assessed by basic local alignment search tool (BLAST) in GenBank database. The species of samples were identified by the sequences with the most similarity. Results showed that the Pheretima’s specific primers MF2R2, Hirudo’s specific primers WF1R2 and WF2R2, Scorpio’s specific primers SF1R1 and SF2R4, could specifically identify the corresponding animal drug that the company used in NXT, namely, *Metaphire vulgaris* (or *Metaphire guillelmi*), *W. pigra* and *M. martensii*, with amplicons of around 230 bp, 200 bp, 250–260 bp, respectively. This DNA molecular identification method is accurate, simple, with high specificity and sensitivity, thus it could be used as a supplement to the conventional method for origin identification and further improve the quality control of three zoological drugs in NXT.

### 3.2 Content determination

In the Chinese Pharmacopoeia of 2020 edition, HPLC based quantitative determination is applied to monitor the paeoniflorin and salvianolic acid B, which are derived from Paeoniae Radix Rubra and Salviae Miltiorrhizae Radix et Rhizoma. According to the regulations, the content of paeoniflorin and salvianolic acid B should not be less than 0.40 mg per capsule, respectively. To date, plenty of studies focus on the content determinations in NXT (details in Table 2) and a mass of active ingredients have been detected, mainly including flavonoids, organic acids, and lactones. Moreover, the trace elements in NXT also have been quantified.

**TABLE 2:** Reported determination methods of chemical components in NXT.

Compounds	Determination methods					References
	Instruments	Chromatographic columns	Mobile phases	Flow rates	Detection wavelengths	
Hydroxysafflor yellow A^(a)^, Paeoniflorin^(b)^, Ferulic acid^(c)^, Salvianolic acid B^(d)^, Kaempferol^(e)^, Formononetin^(f)^, Tanshinone IIA^(g)^	HPLC-DAD	Capcell PAK C_18_ column (250 mm × 4.6 mm, 5.0 μm)	methanol-acetonitrile (25:75)-0.1% formic acid aqueous solution (gradient elution)	1.0 mL/min	240 nm^(b) (f)^, 280 nm^(d) (g)^, 316 nm^(c)^, 403 nm^(a) e)^	Zhang et al. (2019a)
Astragaloside^(h)^, Calycosin-7-glucoside^(i)^, Salvianolic acid B^(j)^, Hydroxysafflor yellow A^(k)^	HPLC-DAD	KromasilC_18_ column (250 mm × 4.6 mm, 5.0 μm)	acetonitrile-0.6% phosphoric acid aqueous solution (gradient elution)	0.8 mL/min	203 nm^(h)^, 322 nm^(i) j) k)^	Su et al. (2015b)
Hydroxysafflor yellow A^(l)^, Paeoniflorin^(m)^, Salvianolic acid B^(n)^, Ferulic acid^(o)^, Ligustilide^(p)^	UPLC-DAD	BEH C_18_ column (2.1 mm × 100 mm, 1.7 μm)	acetonitrile-0.5% formic acid (gradient elution)	0.3 mL/min	400 nm^(l)^, 235 nm^(m)^, 280 nm^(n)^, 324 nm^(o) p)^	Li et al. (2013)
Protocatechualdehyde^(q)^	HPLC-DAD	Linksil-ODS C_18_ column (250 mm × 4.6 mm, 5.0 μm)	acetonitrile-1% glacial acetic acid aqueous solution (isocratic elution)	0.8 mL/min	280 nm^(q)^	Chen et al. (2015)
Paeoniflorin^(r)^	HPLC-DAD	Shim-packCLC-ODS C_18_ column (250 mm × 4.6 mm, 5.0 μm)	methanol-water (isocratic elution)	1.0 mL/min	230 nm^(r)^	Zhang et al. (2010)
Tanshinone IIA^(s)^	HPLC-DAD	ZOR-BAX Eclipse XDB-C_18_ column (250 mm × 4.6 mm, 5.0 μm)	methanol-water (isocratic elution)	1.0 mL/min	270 nm^(s)^	Zhang (2008)
Danshensu^(t)^	HPLC-DAD	Shim-packVP-ODS C_18_ column (250 mm × 4.6 mm, 5.0 μm)	methanol-0.5% glacial acetic acid aqueous solution (isocratic elution)	0.8 mL/min	280 nm^(t)^	Wang (2008)
Astragaloside Ⅳ, Astragaloside Ⅱ, Isoastragaloside Ⅱ, Astramembrannin Ⅱ, Astragaloside Ⅰ, Isoastragaloside Ⅰ	HPLC-ESI/TOF MS	Zorbax Extend C_18_ column (250 mm × 4.6 mm, 5.0 μm)	0.1% formic acid aqueous solution- acetonitrile (gradient elution)	1.0 mL/min	-	Wang et al. (2006)
Paeoniflorin, Ecdysterone, Amygdalin, Mulberroside A, Caffeic acid, Ferulic acid, Salvianolic acid B, Astragaloside IV, Formononetin, Cryptotanshinone, Tanshinone IIA	LC-MS/MS	Eclipse plus C_18_ column (100 mm × 4.6 mm, 1.8 μm)	acetonitrile- formic acid aqueous solution (gradient elution)	0.3 mL/min	-	Li et al. (2017)
Gallic acid, Danshensu, Hydroxysafflor yellow A, Chlorogenic acid, Amygdalin, Protocatechuic aldehyde, (−)-epicatechin, Caffeic acid, Albiflorin, Ononin, Paeoniflorin, Rutin, Salvianolic acid A, Cinnamic acid, Formononetin, Dihydrotanshinne I	RRLC–MS/MS	Eclipse XDS C_18_ column (100 mm × 4.6 mm, 1.8 μm)	acetonitrile- formic acid aqueous solution (gradient elution)	0.3 mL/min	-	Xu et al. (2016b)
Sodium (Na), Calcium (Ca), Magnesium (Mg), Kalium K), Iron (Fe), Chromium (Cr), Manganese (Mn), Nickel (Ni), Cadmium (Cd), Copper (Cu), Zinc (Zn)	atomic absorption spectrophotometer	-	-	-	-	Dong et al. (1997)

## 4 Pharmacokinetics

Pharmacokinetics plays a vital role in drug development and efficacy evaluation. Pharmacokinetics mainly investigate the characteristic interactions of a drug and the body in terms of absorption, distribution, biochemical conversion (or metabolism) and excretion, as well as the dynamic changes of blood concentration. For most TCM, the active ingredient needs to be absorbed into blood and reach the target organs thus exert pharmacological effects. Hence, clarifying which compounds have distinct exposure in blood or tissue after administration is the primary issue when conducting pharmacokinetic researches of TCM.

To date, a series of pharmacokinetic studies of NXT have been carried out. Li et al. identified eleven components in the plasma of the rats orally administered NXT, including tanshinone IIA, ecdysterone, mulberroside A, amygdalin, salvianolic acid B, caffeic acid, astragaloside IV, ferulic acid, formononetin, cryptotanshinone, and paeoniflorin. In addition, the pharmacokinetics of ferulic acid, caffeic acid, formononetin, cryptotanshinone and tanshinone ⅡA in rat plasma were studied. It was found that caffeic acid, ferulic acid and formononetin were rapidly absorbed into the blood of rats after an oral administration of NXT, whose time to reach maximum drug concentration (*T*_max_) were all less than1hour (Li J. et al., 2017). In another study, with the method of everted gut sacs, Yang et al. investigated the absorption of chemical components of NXT in different intestinal segments and found that paeoniflorin, hydroxysafflor yellow A, salvianolic acid B and ferulic acid were absorbed in the ileum and jejunum segments, and the absorption of these four components did not reach *T*_max_ within 3 hours, while the mode of transport of these four components remains to be further studied (Huang et al., 2013). Our group systematically studied the metabolism of NXT in beagle dog by UFLC-Q-TOF-MS/MS. As a result, a total of 25 prototype and 15 catabolites of NXT were detected in beagle dog plasma (He et al., 2021), while 36 prototype and 52 metabolites were identified in feces and urine (He et al., 2019). Meanwhile, the pharmacokinetic profiles of six crucial components including cryptotanshinone, ferulic acid, paeoniflorin, prunasin, senkyunolide G, and tanshinone IIA were investigated and the *T*_max_ of these six compounds were determined as 1.29, 1.12, 2.33, 1.91, 1.25, and 1.75h, while corresponding *C*_max_ were 2.32, 18.99, 41.03, 433.88, 2.76, 2.37 ng/mL, respectively (He et al., 2021). Recently, Li et al. compared the difference of pharmacokinetic parameters of the major bio-active components of NXT in normal and acute blood stasis rats. The result showed that *C*_max_ of ferulic acid and formononetin, AUC_all_ of ferulic acid and ferulic acid, and AUC_INF_obs_ of ferulic acid were significantly decreased in blood stasis rats, suggesting the absorption of the four NXT components was weakened in the pathological state of blood stasis, which might be related to the alteration of hemorheology (Li et al., 2022). The metabolic products are summed in Table 3.

**TABLE 3:** Reported metabolic product in NXT pharmacokinetics study.

Metabolic products	Model species	Biological sample source	References
Tanshinone IIA, Ecdysterone, Mulberroside A, Amygdalin, Salvianolic acid B, Caffeic acid, Astragaloside IV, Ferulic acid, Formononetin, Cryptotanshinone, Paeoniflorin	Rat	plasma	Li et al. (2017)
Paeoniflorin, Hydroxysafflor yellow A, Salvianolic acid B, Ferulic acid	Rat	intestinal sbsorption solution	Huang et al. (2013)
Ferulic acid, Formononetin, Caffeic acid, Tanshinone IIA	Rat	plasma	Li et al. (2022b)
Danshensu, Hydroxysafflor yellow A, Chlorogenic acid, Amygdalin, Mandelonitrile, Prunasin, Protocatechualdehyde, Albiflorin, Paeoniflorin, Caffeic acid, Rutin, Calycosin-7-O-β-D-glucoside, Ecdysterone, Lithospermic acid, Rosmarinic acid, Ferulic acid, Calycosin, 7,3,4-trihydroxyisoflavon, Astragaloside IV, Cinnamaldehyde, Cinnamic acid, Formononetin, Daidzein, Butylidenephthalide, Astragaloside II, Senkyunolide F, Ligustilide, Senkyunolide G, Cryptotanshinone, Tanshinone IIA	Beagle dog	plasma	He et al. (2021)
Protocatechuic acid, Hydroxysafflor yellow A, Chlorogenic acid, Amygdalin, Mandelonitrile, Prunasin, Protocatechualdehyde, Vanillin, Caffeic acid, Albiflorin, Paeoniflorin, paeoniflorgenin, Calycosin-7-O-β-D-glucoside, Danshensu, Cinnamic acid, Rosmarinic Acid, Ferulic acid, Isoliquiritigenin, Calycosin, 7,3,4-trihydroxyisoflavon, 2,3-Dihydrocalycosin, Ligustilide, Senkyunolide F, Formononetin, Daidzein, Senkyunolide H, Senkyunolide G, Senkyunolide B, Carnosic acid, Senkyunolide A, Cryptotanshinone, Tanshinone IIA	Beagle dog	urine	He et al. (2019)
Protocatechuic acid, Hydroxysafflor yellow A, Chlorogenic acid, Amygdalin, Prunasin, Protocatechualdehyde, Caffeic acid, Albiflorin, Paeoniflorin, paeoniflorgenin, Salvianolic acid F, Calycosin-7-O-β-D-glucoside, Ecdysterone, Cinnamic acid, Rosmarinic Acid, Ferulic acid, Salvianolic acid A, Salvianolic acid B, Lithospermic acid, Calycosin, 7,3,4-trihydroxyisoflavon, Benzoylpaeoniflorin, Astragaloside IV, Astragaloside II, Formononetin, Ononin, Daidzein, Astragaloside I, Tanshinone IIB, Cryptotanshinone, Tanshinone IIA	Beagle dog	feces	He et al. (2019)

Given NXT has been widely used in a co-administered treatment with other prescription drugs, such as dual antiplatelet therapy (clopidogrel and aspirin), statins, or other TCMs in treating CCVD, it is necessary to attach importance to the drug-drug interactions in pharmacokinetic studies. The key enzyme system, CYP450 family, plays a vital role in metabolizing various drugs and the regulation of CYP450 enzymes is a pivotal cause of adverse drug-drug interactions. Chen et al. found that NXT significantly promoted the catalytic activities of CYP2C19 enzyme on drug metabolism in human liver microsomes (Hui, 2011a). Their further study demonstrated that NXT could enhance the promoter activity, expression and metabolic activity of CYP2C19 via pregnane X receptor (PXR) (Sun et al., 2016). And the addition of NXT to clopidogrel exhibited increased antiplatelet effect in patients with CYP2C19∗2 gene mutation after percutaneous coronary intervention (PCI) (Hui, 2011b). Ouyang et al. investigated the potential effects of NXT on the CYP450 activities in rats and evaluated the pharmacokinetics effect of NXT on metoprolol tartrate and found that NXT exerted significant effect on inducing CYP3A4 activity while inhibiting CYP2D6 activity *in vivo*, thus affecting the metabolism of metoprolol tartrate and its metabolites, suggesting the importance to have a widespread concern regarding the combined use of NXT and western medicine clinically especially those metabolized by CYP3A4 and CYP2D6, so as to prevent drug-drug-related toxicity and side effects (Ouyang et al., 2019). The above pharmacokinetic studies laid a foundation for the research of NXT’s pharmacological mechanism and provide important information and scientific basis for further rational clinical application of NXT.

## 5 Pharmacological properties

### 5.1 Atherosclerosis

Atherosclerosis is a profound pattern disease of arteriosclerosis, characterized by a buildup of fibrofatty plaque within the innermost layer of an arterial wall (Tabares-Guevara et al., 2021). These plaques develop to thick and narrow the arterial wall within lesions, reducing the blood flow and oxygen supply. Moreover, a fully developed plaque could rupture, leading to dramatic hemorrhage and blood clot formation. In either case, the artery can be entirely blockage with severe blood flow cutting off. In fact, atherosclerosis results in not only myocardial infarction and stroke but also disabling peripheral artery diseases (Badimon and Vilahur, 2014; Tatsumi and Mackman, 2015).

As for atherosclerotic plaque initiation and progression, low density lipoproteins (LDLs), which take charge of blood cholesterol transport, has been well-characterized as the most profound risk factor. Other risk factors, both for atherosclerosis and its thrombotic complication, includes high levels of circulating cholesterol and triglyceride, aberrant lipid intake and metabolism, obesity, hypertension, cigarette smoking and diabetes mellitus (Yang et al., 2016; Lu et al., 2022). Increasing evidences have demonstrated the potent role of NXT in alleviating hyperlipidemia and excessive LDL particles in plasma, which is a prerequisite for plaque initiation. In high fat diet or genetic modified ApoE knockout mice, 10 weeks of NXT gavage (624 mg/kg bodyweight per day (mpk)) remarkable attenuated the accumulation of total cholesterol (TC), triglyceride (TG), and low-density lipoprotein cholesterol (LDL-C) in circulating blood, while restored the content of serum high density lipoprotein cholesterol (HDL-C) (Yang et al., 2016). In the liver of high-fat diet fed ApoE KO induced atherosclerotic mice, by activating hormone-sensitive lipase (HSL), adipose triglyceride lipase (ATGL) and energy-sensing AMP-activating protein kinase (AMPKα) signaling, NXT downregulated hepatic lipid contents with reduced abundance of TC and TG enriched droplets (Yang et al., 2017).

Atherosclerotic plaque is initiated when circulating LDL particles penetrate and accumulate into the innermost layer, tunica intima, of the arterial wall. This happens when circulating monocytes respond to the Chemoattractant signaling of the inflamed site, recruited into the intima layer where they mature into macrophages that are capable of recognizing and engulfing intima resident LDL particles and turn into foam cells. In the pro-atherogenic model of ApoE^−/−^ mice fed an atherogenic diet, Wang et al. found that 8 weeks of NXT gavage (624 mpk) could significantly decline the accumulation of foam cells in atherosclerotic plaques, which was associated with the reduction of cellular uptake of oxidized LDL (ox-LDL) as well as the decrease of surface expression of scavenger receptor class A (SR-A) and B (SR-B) (Wang et al., 2018). Another study found that NXT downregulated the gene expression of monocyte chemoattractant protein-1, which compromised the penetrate of monocytes into the tunica intima layer and thus alleviated the foam cell formation (Yang et al., 2016; Long-Tao and Chang-Geng, 2018). Using HFD induced atherosclerosis in LDLR deficient mice, Jin et al. reported the inhibitory of NXT on dendritic cell maturation. NXT treatment significantly delayed the maturation of dendritic cells within lesion, as evidenced by reduced abundance of surface molecules CD40, CD86, CD80 as well as compromised production of pro-inflammatory IL-12p70 (Zhao et al., 2013). As dendritic cells actively participate in early foam cell formation, lipid homeostasis as well as efficient T cell activation, this study shed light on the potential effects of NXT in regulating innate-adaptive immune crosstalk during atherosclerotic plaque development.

The most life-threating event in atherosclerosis is the sudden rupture of a vulnerable plaque, which is the leading cause of acute hemorrhage and severe thrombosis. Hence, increasing the stability of vulnerable plaques is of top priority in disease intervention and management (Han et al., 2019; Mushenkova et al., 2020). Vulnerable plaques are often associated with high inflammatory cell content and the presence of a large necrotic core. Within plaques, inflammatory activation of macrophages stimulates the release of pro-inflammatory cytokines, inhibits interstitial collagen synthesis and protease digestion of fiber cap components (Nakajima et al., 2022). Studies demonstrated NXT could help to maintain unstable plaque. In high-fat diet fed apoE (−/−) mice, NXT increased the content of smooth muscle cells/collagen in the cap of aortic wall lesions, while reducing the buried fiber cap, mineralization and macrophage aggregation in the lesions, suggesting that NXT can reduce advanced atherosclerosis while improve plaque stability (Yang et al., 2016).

As atherosclerosis progresses, the unstable plaques may eventually rupture to form arterial thrombi (Han et al., 2019). Such thrombus potentially disrupts or even blocks the blood flow, leading to health-threating complications including myocardial infarction and acute ischemic stroke, depends on different sites of obstruction (Asada et al., 2018; Stark and Massberg, 2021). Currently, the “gold-standard” strategy for thrombotic management is the long-term application of anti-platelet drugs, among which aspirin and clopidogrel are the most widely applied. These two drugs, however, could lead to detrimental consequences. Notably, long-term, even lifelong anti-platelet meditation dramatically increases the risk of accidental bleeding and hemorrhage transformation. Thus, it is of great demand of seeking alternative strategies which can balance the effects between thrombosis and hemorrhage. Using acute stasis rats, we have investigated the antithrombotic effect of NXT *in vivo*. Compared with model group, NXT treatment indicated a potent improvement of blood coagulation and platelet aggregation, as evidenced by decreased the whole blood viscosity at low shear rates, prolonged prothrombin time and activated partial thromboplastin time, decreased the fibrinogen (FIB) level, and reduced the level of platelet activating factor (Zhang et al., 2020b). Thrombin and factor Xa are two critical modulatory targets of anticoagulants. Recently, Du et al. reported that NXT extract exhibited great activity against thrombin and factor Xa. Six feature components of NXT extract were screened out, namely, paeoniflorin, lithospermic acid, salvianolic acid B, Z-ligustilid, 3,5-dicaffeoylquinic acid, rosmarinic acid, which accounted for the predominant inhibitory activities of thrombin/factor Xa (Du et al., 2022). Based on the carrageenan-induced mouse model, Yang et al. found that NXT treatment could reduce thrombi, which was related to the downregulation of serum TNF-α and P-selectin levels. *In vitro*, NXT effectively reduced lipopolysaccharide-activated adhesion of THP-1 monocytes to human umbilical vein endothelial cells HUVECs, and inhibited oxidized LDL activated platelet adhesion. Of note, NXT administration could antagonize clopidogrel induced cell death in HUVECs, which shed light on the potential of NXT in combination therapy to recover clopidogrel caused cellular damages (Li et al., 2018). Moreover, using a thrombosis mode in zebrafish, Wang et al. demonstrated the attenuation of NXT treatment in zebrafish tail venous thrombus with enhanced quantity of heart red blood cells through modulating COX2-VEGF/NFκB signaling (Wang et al., 2021).

The role of the gut microbiota in atherosclerosis has begun to be appreciated in recent years. Mounting evidence has indicated the imbalanced gut microbial community in humans is associated with the incidence of atherosclerosis (Jonsson and Bäckhed, 2016). Recent studies have convincingly linked gut microbiota to traits relevant to atherosclerosis, such as dyslipidemia, inflammation and insulin resistance (Org et al., 2015). It is noteworthy that a series of recent studies demonstrated that NXT could alleviate atherosclerotic cardiovascular diseases partially by regulating the structure of gut microbiota. With the high-fat diet (HFD)-fed minipig model, we found that the long-term administration of NXT could regulate blood lipid profiles, inhibit vascular inflammation, enhance antioxidant capacity, and alleviate myocardial injury. NXT could increase the diversity of gut microbiota and reverse the increase of Firmicutes/Bacteroidetes (F/B) ratio caused by high fat diet. Some key bacterium, including *Caproiciproducens*, *Sutterella*, Erysipelotrichaceae, and *Romboutsia* were found to be closely involved in NXT’s effects (Zhang et al., 2019). In another experiment, Li et al. explored the preventive effect of NXT on hyperlipidemia in HFD-fed rat model and also investigated the regulation of gut microbiota, and found that NXT could significantly reduce TC, TG, LDL-C levels and increase HDL-C level in serum. And NXT significantly altered the composition of gut microbiota and changed the fecal levels of gut microbiota related metabolites, including bile acids (BAs) and short chain fatty acids (SCFAs). Some important factors as F/B ratio, the amount of propionic acid and the relative abundance of *Collinsella* were proposed to be potential therapeutic biomarkers (Lu et al., 2022).

Statins are the first-line lipid-lowering drugs used clinically for the treatment of atherosclerosis. However, long-term statin therapy often causes side effects such as liver injury and rhabdomyolysis. NXT combined with statins not only have beneficial effects on atherosclerosis, but also can reduce the adverse effects of statins. Yang et al. suggested that the combination of NXT and atorvastatin could, on one hand, enhance plaque stability and, on the other hand, attenuate atorvastatin-induced acute liver inflammation (Yang et al., 2017). In addition, we have demonstrated that NXT administration could attenuate atorvastatin induced excessive oxidative stress, hepatic and renal dysfunction, inflammatory activation, and endothelial integrity (Zhang et al., 2020a). These drug combination studies suggest that the co-treatment of NXT to atorvastatin can enhance safety and efficacy.

### 5.2 Cardiovascular disease (CVD)

Cardiovascular disease (CVD) is a category of diseases associated with heart or cardiovascular vessels, including coronary artery diseases, myocardial infarction, post-infarction myocardial remodeling, and cardiomyopathy (Wang et al., 2021). The World Health Organization defines cardiovascular disease as a leading life-threaten cause globally. In 2016, there was an estimated of 17.9 million cardiovascular disease induced death, accounted for 31 percentiles of all-cause mortality globally (Mendis et al., 2015). Among CVD, myocardial infarction has become one of the leading causes of death in the world. It is caused by myocardial ischemia and hypoxia due to coronary artery disease. Myocardial infarction happens when blood flow decreases or even stops, leading to dramatically myocardial ischemia and tissue death. Besides the beneficial outcomes of NXT in treating thrombosis, its cardioprotective effects against myocardial infarction had been systematically explored and highlighted. In one study, using cardiogenic cell line H9c2, Xu et al. suggested the water exact of NXT could significantly protect against superoxide induced cell death via modulating PPARα expression, and promote autophagy and autophagy associated genes, leading to inhibition of mTOR signaling (Xu et al., 2016). Moreover, NXT water extract could potently alleviate cardiomyocyte hypertrophy in H9c2 cells, such effect is achieved, at least in part, through regulating PPAR𝛾-mediated autophagy (Yuan et al., 2017). In another study, the *in vivo* intestinal absorption liquid was employed instead of drug containing serum or extraction fractions and found that precondition of NXT remarkably enhanced the anti-oxidative capacity, inhibited activation of PPAR/caspase-3, rescued mitochondrial membrane potential, and reduced intracellular Ca^2+^, thereby protecting myoblasts from oxidative injuries and programmed cell death (Zhang et al., 2013). Recently, Wu et al. assessed the therapeutic outcomes of NXT on acute myocardial infarction induced by ligation of the left anterior descending coronary artery in rats and investigated the pharmacodynamic mechanisms from the perspective of metabolic reprogramming. After NXT treatment, heart function was significantly recovered, with both left ventricular ejection fraction (LVEF) and left ventricular fraction shortening (LVFS) improved. Histology revealed decrease inflammatory infiltration, alleviated myocardial fibrosis, and reduced infarct size in NXT treated rats. Targeted metabolomics further demonstrated NXT treatment could restore myocardial ischemia induced metabolic rewiring, including glycolysis, tricarboxylic acid (TCA) cycle, oxidative phosphorylation, purine metabolism and glutathione metabolism. In line with shifted metabolite profiles, mechanism studies indicated the effects of NXT in regulating metabolism associated crucial proteins, including silent information regulator 1 (SIRT1), peroxisome proliferator-activated receptor gamma coactivator 1α (PGC-1α), and a subunit of mitochondrial ATP synthase (ATP5D). These results together emphasized the cardioprotective effects of NXT against myocardial infarction, and highlighted the regulatory roles of NXT in energy metabolism related metabolic reprogramming (Zhao et al., 2022). Activated platelets is another crucial risk factor for myocardial infarction occurrence. A platelet-fibrin plug often forms at the site of ruptured atherosclerotic plaque, which could lead to myocardial infarction (Oikonomou et al., 2020). Study of Wang et al. revealed that NXT significantly decreased infarct size, decreased lactic dehydrogenase (LDH), creatine kinase (CK), and creatine kinase-MB (CK-MB) values, and improved cardiac function in rats with myocardial infarction. Such beneficial effects were achieved, at least in part, by inhibiting platelets activation via down-regulating ROS levels, decreasing ERK5 phosphorylation, and increasing RAC1 phosphorylation (Zhang et al., 2022).

### 5.3 Cerebrovascular disease

The brain is one of the most important and subtle organs in the human body, and the cerebral circulation continuously supplies blood to the brain. All diseases affecting brain circulation can be defined into the category of cerebrovascular diseases, with stroke being the deadliest, accounting for more than 10% of all cause deaths worldwide. Cerebral ischemia is caused by embolic occlusion or thrombotic of a cerebral artery, accounting for more than 60% of stroke cases (Collaborators, 2021). On one hand, under ischemic stroke, the blood supply to part of the brain is compromised and oxygen is deprived, often leads to neuronal cell death and functional losses. When the blood supply is restored, on the other hand, reperfusion injury occurs because of increased production of reactive oxygen/nitrogen species, microvascular vasoconstriction as well as cytokine release. NXT has been shown to have a beneficial effect on ischemic stroke. Using a middle cerebral artery occlusion (MCAO) rat model, Yang et al. conducted a UPLC/TOF-MS-based metabolomic study to explore potential biomarkers in plasma associate with cerebral ischemia as well as evaluated the therapeutic effects of NXT and found that NXT treatment could significantly improve neurological deficits and reduce cerebral infarct size. Meanwhile, NXT treatment obviously altered the levels of the potential therapeutic targets, such as glutamine, PE (17:0), LysoPE (20:1), LysoPE (24:0), etc., suggesting that the therapeutic effects of NXT on cerebral ischemia are partially due to interference with glutamine metabolism and lipid metabolism (Liu et al., 2016). Amino acids in cerebrospinal fluid perform critical roles in cerebral ischemia. Recently, focusing on the amino acids in cerebrospinal fluid, the same group conducted an amino acid-protein interaction imbalanced network of cerebral ischemia to further investigated the pharmacological mechanisms of NXT. They observed the dynamic levels of amino acids in cerebrospinal fluid 3,6,12, and 24 h after MCAO, and found that NXT treatment could restore the abnormal level of nine amino acid biomarkers at 24 h after MCAO, including alanine, valine, taurine, leucine, isoleucine, glutamine, glutamic acid, phenylalanine, (S)-tyrosine, and tryptophan. And, aromatic-L-amino-acid decarboxylase (AADC) was found to be one of the putative targets for NXT in the protection against cerebral ischemia (Xu et al., 2019). In a permanent MCAO model, Li et al. conducted combining LC–MS/MS and transcriptome microarray analyses to investigate the protein and mRNA profiles and the changes in the rat cerebral cortex during cerebral ischemic injury and repair processes due to NXT treatment. They have identified 39 potent candidates, signaling pathways and core markers for cerebral ischemic injury modulated by NXT, among which marked MAPK signaling as a major target for NXT (Liu et al., 2020). Using a cerebral ischemia-reperfusion (I/R) injury mice model, Xue et al. evaluated the neuroprotective effect of NXT against I/R as well as relevant mechanism. Compared with model group, NXT treatment had significantly improved neurological defect, reduced infarct volume and brain edema. Meanwhile, NXT downregulated the elevated levels of MDA and SOD. The neuroprotective mechanisms of NXT against cerebral I/R injury may associated with the anti-oxidant and anti-inflammation activities via decreasing the expression of LOX-1, phosphorylation of ERK1/2 and translocation of NF-κB p65 (Xue et al., 2016).

Ischemic stroke, hemorrhagic stroke, hypoxic-ischemic brain injury and other cerebrovascular diseases can cause insufficient blood flow, leading to functional impairment and cognitive defects of the brain, such as vascular dementia (VD). Ma et al. explored the role of NXT on VD in mice model with chronic hypoperfusion damage, which was established by double carotid artery coarctation operation. NXT could recover the loss of space learning and memory, improve the morphological alterations of neural tissues, inhibit the activation of pro-inflammatory astrocytes, and eventually rescue the ischemic damages of the brain (Ma et al., 2015). Other studies revealed that NXT could protect against VD by enhancing hippocampus VEGF expression, inhibiting endothelial apoptosis, sustaining endothelial barrier integrity, promoting angiogenesis (Wei et al., 2006) and regulating choline acetyl transferase in cholinergic neural circuits (Sun and Zeng, 2022). Moreover, in APPswe/PSEN1dE9 double transgenic (APP/PS1) mice, the beneficial outcomes of NXT against cognitive deficit had been further clarified. NXT inhibited neuron atrophy and apoptosis by reducing the contents of pro-inflammatory mediators including IL-1β, IL-6, TNF-α in the brain. Meanwhile, in a mouse hippocampus neuronal cell line HT-22, NXT had been found to mitigate L-glutamic acid enhanced inflammation, reactive oxygen species (ROS) production and apoptosis, partially via down-regulating TLR4/NF-κB/IL-1β pathway (Wang et al., 2021). These studies together emphasize NXT as a promising candidate for treating cerebral vascular diseases as well as vascular associated cognitive disorders.

### 5.4 Other diseases/disorders

In addition to cardiovascular and cerebrovascular diseases, other diseases can also be improved by NXT treatment, all of which are reported to be related to diabetes. Diabetes is characterized by long-term hyperglycemia, which can cause multiple complications. In past years, our group first combined the gut microbiome and metabolomics to explore the effects and potential mechanisms of NXT in anti-diabetes. Based on the type 2 diabetes mellitus (T2DM) rat model established by HFD combined with streptozotocin (STZ) injection, 8 weeks of NXT gavage (1,000 mpk) was found to significantly ameliorate hyperlipidemia and hyperglycemia, improve insulin resistance, reduce inflammation and alleviate myocardial injury. Meanwhile, NXT recovered the richness and diversity of microbial community in T2DM rats. At genus, NXT significantly altered the relative abundances of certain gut bacteria, including *Erysipelatoclostridium*, *Oscillibacter*, *Ruminiclostridium* 9 and *Ruminococcus* 1. Besides, NXT significantly changed the levels of metabolites in serum, mainly including fatty acids, lysophospholipids, bile acids and other metabolites. Several feature pathways, such as arachidonic acid metabolism, fatty acid β-oxidation and glycerophospholipid metabolism, were identified as the key pathways of NXT in anti-diabetes (Yan et al., 2020).

Diabetic nephropathy is one of the complications of diabetes with high morbidity and mortality. Using 6-week-old adolescent db/db mice, Yang et al. first evaluated the protective effects of NXT on diabetic nephropathy, and found NXT significantly reduced diabetes-increased glucose levels, ameliorated serum lipid profiles, and improved renal functions (Yang et al., 2018). Recently, in 12-week-old adult db/db mice, they further explored the therapeutic outcomes of NXT. At late phase of diabetic nephropathy development, NXT treatment was also capable of reducing diabetes-induced systematic hyperglycemia and dyslipidemia. Moreover, after NXT post-treatment, several feature symptoms including high secreted albumin and ratio of albumin to creatinine in urine, kidney atrophy/hypernephrotrophy, mesangial matrix expansion as well as glomerulosclerosis are all remarkably attenuated. Such protective effects were associated, at least in part, with MMP2/9 activation, inactivation of TGF-β1 pathway, inhibitory upregulation of adhesion molecules in the kidney as well as activation of AMPKα-mediated glucose and energy metabolism in liver, adipose tissue, and skeletal muscle (Yang et al., 2019).

**FIGURE 2:**
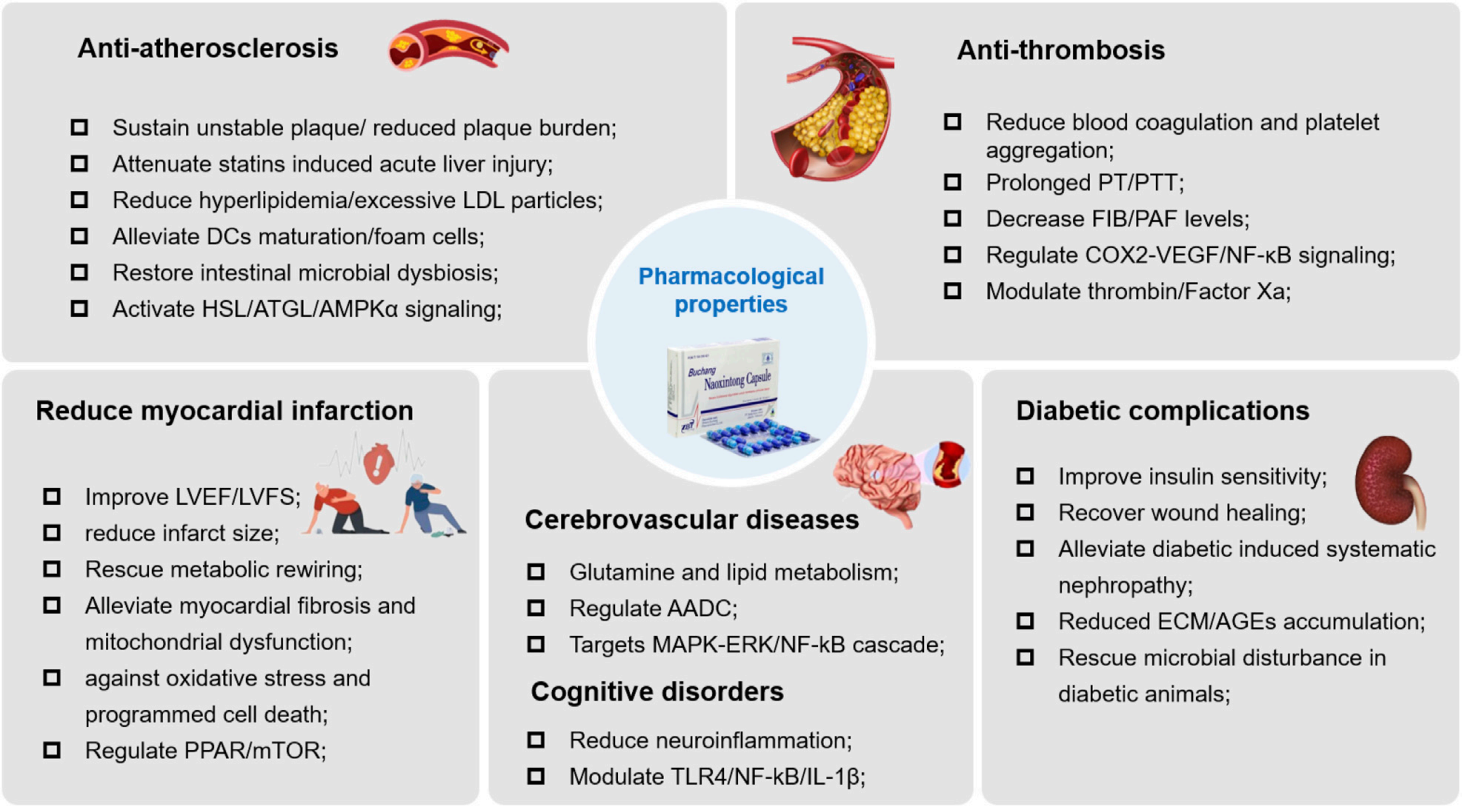
The pharmacological properties of NXT.

Diabetic wound healing is a troublesome in diabetes mellitus. And, nearly one-third of costs are attributed to the management of wound healing associated diabetic foot disorders (Driver et al., 2010). To this concern, effective strategies are urgently needed to mitigate the incidence and severity of these chronic, non-healing diabetic foot ulcers. In one study, Fang et al. investigated the effects of NXT in a murine model of diabetic wound healing and found that NXT could accelerate diabetic wound closure, accompanying by the upregulation of ECM remolding and collagen deposition. For mechanism, NXT promoted neutrophils efferocytosis, which might help to alleviate neutrophils induced tissue damages (Fang et al., 2021). In addition, NXT had been reported to rewire the polarization of M2 macrophages through regulating JAK/SATA cascades, which would facilitate focal inflammatory resolution and macrophage mediated tissue repair (Fang et al., 2021). These results, when taken together, emphasize the promising potential of NXT as a combination therapeutic strategy in the management of diabetic complications. The pharmacological properties of NXT were illustrated in Figure 2.

### 5.5 The brief summary for the co-treatment of CCVD

So far, many studies, both *in vitro* and *in vivo*, have proven the beneficial effects of NXT in the prevention and treatment of CCVD in varied pathological stages. These studies have been carried out in various animal models as well as cell models, providing considerable evidences in both pharmacological and mechanism perspective. Multiple protective effects of NXT on CCVD have been reported, including atherosclerosis, thrombosis, myocardial infarction, ischemic stroke, and diabetic nephropathy. Biologically, these protective effects on cardio-cerebrovascular diseases of NXT can be associated with its actions on lipid/glucose metabolism, inflammation, oxidative stress, apoptosis and gut microbiota. As the representative prescription of co-treatment of CCVD, NXT possesses complex components and plays a therapeutic effect through multiple targets and multiple pathways. The pharmacological properties of main active components of NXT are summarized in Table 4. In brief, NXT performed its therapeutic effect on the co-treatment of CCVD through the pattern “multiple components-multiple targets-multiple pathways”.

**TABLE 4:** The pharmacological properties of main active components of NXT.

Component	Pharmacological properties	Functions/mechanisms involved	References
Astragaloside Ⅳ	Alleviates atherosclerosis, acute myocardial infarction, ischemic stroke and diabetes	Anti-inflammation, antifibrotic, anti-oxidative stress, immunoregulation	Li et al. (2017b)
Danshensu	Alleviates atherosclerosis, acute myocardial infarction, myocardial I/R injury	Anti-inflammation, inhibition of reactive oxygen species (ROS) production	Lin et al. (2020)
Caffeic acid	Alleviates diabetes and associated complications	Anti-inflammation, anti-oxidative stress, immune-stimulatory	Ganguly et al. (2023)
Tanshinone ⅡA	Attenuates atherosclerosis, acute myocardial infarction, myocardial I/R injury, cerebral I/R injury	Anti-inflammation, anti-oxidative stress, inhibition of apoptosis	Chen et al. (2020), Chang et al. (2024)
Rosmarinic acid	Attenuates diabetes and associated complications	Reduction of oxidative and inflammatory damage, hyperinsulinism, insulin resistance	Ekbbal et al. (2023)
Cryptotanshinone	Attenuates diverse liver diseases	Reduction of hepatic oxidative stress, inflammation, and apoptosis	Hwang et al. (2023)
Salvianolic acid B	Ameliorates myocardial I/R injury	Anti-inflammation, anti-apoptosis, anti-oxidation	Wang et al. (2013)
Protocatechualdehyde	Ameliorates cerebral I/R injury	Inhibition of ROS production	Duan et al. (2017)
Ferulic acid	Attenuates atherosclerosis, and hypertension	Anti-inflammation, anti-oxidative stress	Srinivasan et al. (2007)
Hydroxysafflor yellow A	Ameliorates acute myocardial infarction, ischemic stroke and hypertension	Anti-inflammation, anti-apoptosis, inhibition of ROS production, promotion of angiogenesis	Ao et al. (2018)
Ligustilide	Alleviates acute myocardial infarction	Anti-inflammation, inhibition of ROS production	Choi et al. (2018)
Amygdalin	Alleviates atherosclerosis and ischemic stroke	Anti-inflammation	Lv et al. (2017)
Paeoniflorin	Alleviates atherosclerosis, acute myocardial infarction and ischemic stroke	Activation of lipid metabolism, anti-inflammatory, anti-apoptosis and inhibition of ROS production	Yu et al. (2013)
Gallic acid	Ameliorates acute myocardial infarction and hypertension	Anti-inflammation, inhibition of ROS production; promotion of lipid metabolism; attenuation of vascular calcification	Jin et al. (2017), Choubey et al. (2018)

Actually, cardio-cerebrovascular diseases are commonly caused by the abnormality of “blood” and “blood vessel”, that is, the abnormal change of hemorheology, including the blood viscosity, blood coagulation, etc. and the change of vascular morphology and biologic functions. Modern medicine believes that atherosclerosis is the common pathological basis of cardio-cerebrovascular diseases. From this point of view, the common underlying mechanism of NXT for the co-treatment of cardio-cerebrovascular diseases has been discussed in detail in section 5.1. However, given that these effects are scattered throughout the course of CCVD, randomized controlled trials with long-term follow-up are urgently needed to clarify the precise roles of NXT in personalized medicine and to declare its rationality in combination therapy.

## 6 Clinical research

### 6.1 Cardiovascular disease

#### 6.1.1 Acute coronary syndrome (ACS)

ACS is the leading cause of death in coronary heart disease (CHD), often caused by the instability of atherosclerotic plaque (Zhang et al., 2023). The mainstay treatment for ACS is percutaneous coronary intervention (PCI), which can quickly reconstitute the blood supply and effectively remove vascular stenosis or occlusion (Dong et al., 2023). However, PCI may cause plaque rupture, platelets activation, formation of blood clots and could even lead to a secondary thrombus (Abbate et al., 2012). And, dual antiplatelet therapy (aspirin plus clopidogrel) is the most commonly treatment strategy for ACS patients undergoing PCI. It is worth mentioning that the antiplatelet effect of clopidogrel depends primarily on its activation by the cytochrome P450 (CYP) system, particular CYP2C19 enzyme. The person who has more CYP2C19*2 copies possesses reduced CYP2C19 enzyme activity will be less sensitive to clopidogrel treatment due to the poor metabolism of clopidogrel (Fontana et al., 2008). In many cases, dual antiplatelet therapy does not achieve the ideal therapeutic effect in patients who carried the CYP2C19∗2 polymorphism as the part of clopidogrel does not work well. Interestingly, about 50% population in China possesses CYP2C19*2 the nonfunctional allele (Fontana et al., 2008), which means that the clopidogrel therapy may does not work well in half of Chinese patients. In China, to further enhance the clinical efficacy, some TCMs will be used as the secondary prevention in addition to aspirin and clopidogrel. In the study of Chen et al., ninety patients with CYP2C19*2 polymorphism were randomly assigned to receive either adjunctive NXT (triple group, 45 cases) or dual antiplatelet therapy (dual group (aspirin plus clopidogrel), 45 cases). The authors compared the degree of platelet inhibition at 7 days after treatment and the rate of subsequent major adverse cardiovascular events (MACEs) during a 12-month follow-up. They found that the adjunctive NXT to dual antiplatelet therapy could significantly enhance the antiplatelet effect and decrease subsequent MACE in patients with the CYP2C19*2 polymorphism undergoing PCI(Chen et al., 2014). Despite the sample size in the study was small, the positive results in long follow-up periods (12-month) makes the claim convincing that the addition of NXT to dual antiplatelet therapy helped a lot in patients with the CYP2C19*2 polymorphism. The specific mechanism of action needs further study.

In recent years, NXT has also been reported effective when combined with Danhong injection in ACS patients undergoing PCI base on the secondary prevention. Both of the two clinical trials revealed that the combination of NXT and Danhong injection had better effects on cardiac functions than each of them alone (Lv et al., 2015; Zhao et al., 2018). Thereinto, one study is a multicentered, randomized controlled, double-blind trial, placebo-controlled, and parallel-designed clinical trial with 65 cases in each group. The primary outcomes were clinical curative effects and cardiovascular events. The secondary outcomes included parameters from laboratory examinations: plasma ET-1, NO, VWF, and cardiac function such as left ventricular ejection fraction (LVEF). In the NXT combination group, the authors observed the better signs of clinical efficacy, such as reduced LVEF, higher nitric oxide levels, and lower levels of endothelin-1 (ET-1) and von Willebrand factor (VWF), without detectable cardiovascular events or other adverse reactions. Another study investigated the effect of NXT combination on short-term prognosis by comparing the incidences of major adverse cardiovascular events and cardiac functions, including ejection fraction and 6-min walk test distance, and serum level of the ACS marker (sCD40 L), during hospital discharge and at 3 months after PCI. The conclusion is that the NXT combination exerted significant effect in improving the cardiac functions at 3 months following discharge, but not at the time of discharge. However, some limitations did still exist in both two studies. For instance, they did not examine end points such as all-cause mortality, the sample size was small, and the follow-up time of 3 months is relatively short.

Systematic review and meta-analysis provide an available approach to assess the existing evidence to support the clinical practice. Recently, a meta-analysis of 13 clinical randomized trials (RCTs) involving 1,286 patients after PCI revealed that compared with the control group treating with conventional WM, NXT combined WM group showed better efficacy in decreasing the maximum platelet aggregation rate, lowering the levels of plasma vWF, GPⅡb/Ⅲa, and fibrinogen, and reducing the occurrence of adverse cardiovascular events (Liu et al., 2022). However, because of the limited number and quality of articles included, the findings of meta-analysis need to be validated by additional more high-quality, and double-blinded multicenter RCTs with a larger sample size in the future. Taken together, to some extent, the positive results in clinical trials suggested that NXT could be one of the complimentary drugs used in the secondary prevention for ACS patients after PCI.

#### 6.1.2 Chronic stable angina (CSA)

CSA, characterized by chest pain or discomfort, is a major symptomatic presentation in about 50% of patients with CHD (Gao et al., 2021). In China, the prevalence rate of CSA was about 3.6% in 2017 and the numbers are still rapidly increasing in recent years due to ageing as well as multiple risk factors such as diabetes mellitus, hypertension, etc., (Wang et al., 2022). Currently, guideline-directed therapy for CSA mainly includes antiplatelet agents, statins, calcium channel blockers and nitrates (Healthcare Engineering, 2023). However, some adverse reactions of the long term anti-atherogenic interventions have been reported (Banach and Mikhailidis, 2018; Qian et al., 2021). Besides, a number of patients are less sensitive to the antiplatelet treatment. Recently, in a clinical study by Qiu et al., NXT (4 capsules/time, 3 times per day) plus ticagrelor showed better total effective rate (92.16%) compared with ticagrelor alone (76.47%). And, NXT combination can better lower the frequency and duration of angina pectoris and reduce the serum cardiac troponinⅠ (cTnⅠ), CK-MB, myoglobin (MYO), myeloperoxidase (MPO) and lipid peroxide (LPO) levels, while elevate the left ventricular ejection fraction (LVEF) and stroke-volume (SV), suggesting that adding NXT to ticagrelor helped a lot in patients with CSA, especially in improving cardiac function and reducing angina pectoris symptoms (Qiu et al., 2023). Though the difference of curative effect between NXT combination and ticagrelor was significant and clear, the study has some flaws in the study design. For instance, this is a small scale RCT study (51 case in each group), with only 8-week intervention period, which is relatively short, and without follow-ups, making it less convincing. It is worth mentioning that CSA affects a large population across the world and has a high mortality rate. Therefore, a large-scale and long-term future study with a longer-term follow-up that assesses mortality as the primary endpoint is expected.

### 6.2 Cerebrovascular disease

#### 6.2.1 Ischemic stroke

In the past nearly 30 years from 1990 to 2019, at least 10% global population died from ischemic stroke, accounting for half of all deaths in cerebrovascular disease (Group et al., 2020). The main treatment methods of ischemic stroke include craniotomy, minimally invasive intervention and drug therapy. However, both of craniotomy and minimally invasive intervention can be extremely traumatic, which should only be used as a last resort. Pharmacological intervention is the most conventional option, such as aspirin and clopidogrel are often used to treat IS but which can lead to an increased risk of bleeding in the brain. NXT is reported to be promising as a new alternative treatment. There have been several reviews demonstrated the beneficial effect of NXT on the treatment and prognosis of ischemic stroke (Li et al., 2021; Li et al., 2021). A considerable number of clinical studies have also been conducted on the treatment of ischemic stroke by NXT. In a clinical trial study, 153 patients (>65 years old) with non-valvular atrial fibrillation (NVAF) and genetic variants of vitamin K epoxide reductase (VKORC1) were selected to receive oral aspirin combined with NXT or warfarin and were followed for at least 1 year (Tohgi et al., 1991). The result showed that NXT has similar therapeutic effect but less risk of bleeding and all-cause death during the 1-year follow-up. Although the number of participants in the study was relatively small, the long-term follow-up still revealed part of the efficacy of NXT. Another study which focused on stroke risk in 600 patients with NVAF compared with the general population have shown that the incidence of ischemic stroke in the general population increases with age and has no difference between treated and untreated subjects among hypertensives (Wang et al., 2018b). Therefore, for patients who cannot tolerate warfarin, NXT may be considered as an alternative therapy. Only sufficient amount of data can provide more convincing validation, we need more experiments to verify the efficacy of NXT on patients of different ages.

#### 6.2.2 Vascular dementia (VD)

VD is a group of mental and cognitive dysfunction syndromes caused by cerebrovascular accident and is one of the common causes of senile dementia. Drug therapy is the only treatment for VD. The commonly used drugs include acetylcholinesterase inhibitors, non-competitive N-methyl-D-aspartate (NMDA) receptor antagonists, aspirin, and heparin. Compared with them, NXT has fewer side effects (Kombo et al., 2022). Recently, a meta-analysis study revealed that the use of NXT in the treatment of VD can increase the clinical effect, improve cognitive function, enhance the ability of daily living and reduce the degree of clinical dementia, without increasing the risk of adverse reactions (Li et al., 2022). A total of 33 studies comprising 2,947 patients with VD were included in this study. However, the low methodological quality of the included RCTs that reduced the quality of evidence: first, none of the studies mentioned the allocation hiding and blind method, which had the risk of serious bias; secondly, mini-mental state examination (MMSE)score and activities of daily living (ADL) score index may have heterogeneity due to different treatment course and intervention measures in control group. Finally, the sample size of ADL score index is relatively small which would lead to large error in results. Thus, the findings remain to be questioned. The high-quality multicenter RCTs with more scientific experimental designs to derive more scientific and accurate conclusion are urgent needed.

### 6.3 Diabetes complications

With the prevalence of unhealthy lifestyles, the incidence of type 2 diabetes has been increasing and its related medical burden has been rising accordingly (Guo et al., 2023). In recent years, NXT showed its therapeutic potential in the treatment of diabetes complications in clinic including diabetic nephropathy, diabetic peripheral neuropathy, etc. In a clinical study involving 180 T2DM patients with atherosclerosis, NXT administration (3 capsules/time, 3 times per day) significantly decreased carotid artery intimal medial thickness (IMT) and plaque area, reduced levels of TC, TG, LDL-C, fasting plasma glucose (FPG), glycated hemoglobin A1c (HbA1c), while increased peak systolic velocity (PSV) and end diastolic velocity (EDV) (Cheng et al., 2016). In a clinical observation involving 80 patients, Zhou et al. investigated the effect of NXT (3 capsules/time, 3 times per day) combined with alprostadil in the treatment of diabetic nephropathy. NXT combination significantly decreased the 24 h urinary protein quantification and urinary albumin excretion rate as well as lowered the urea nitrogen and creatinine levels (Zhou et al., 2020). Another clinical study revealed that NXT is capable of improving the therapeutic effects in the treatment of diabetic peripheral neuropathy, such as increasing the motor nerve conduction velocity and sensory nerve conduction velocity (Zhang, 2015). Recently, a real-world retrospective cohort study concerning diabetic kidney disease (DKD) involving 1,798 DKD patients revealed that NXT can slow the decline of estimated glomerular filtration rate and reduce the risk of renal outcomes (Zhang et al., 2022). It is worth mentioning that almost all current RCTs concerning its effect in treating diabetes complications were conducted in China, and the published articles were written in Chinese, which may lead to potential selection bias. Thus, on account of some limitations, more high-quality, and double-blinded multicenter RCTs with a larger sample size are needed to test and verify the positive results of NXT in the treatment of diabetes complications in the future and high quality of clinical evidence is expected.

### 6.4 Other diseases

**FIGURE 3:**
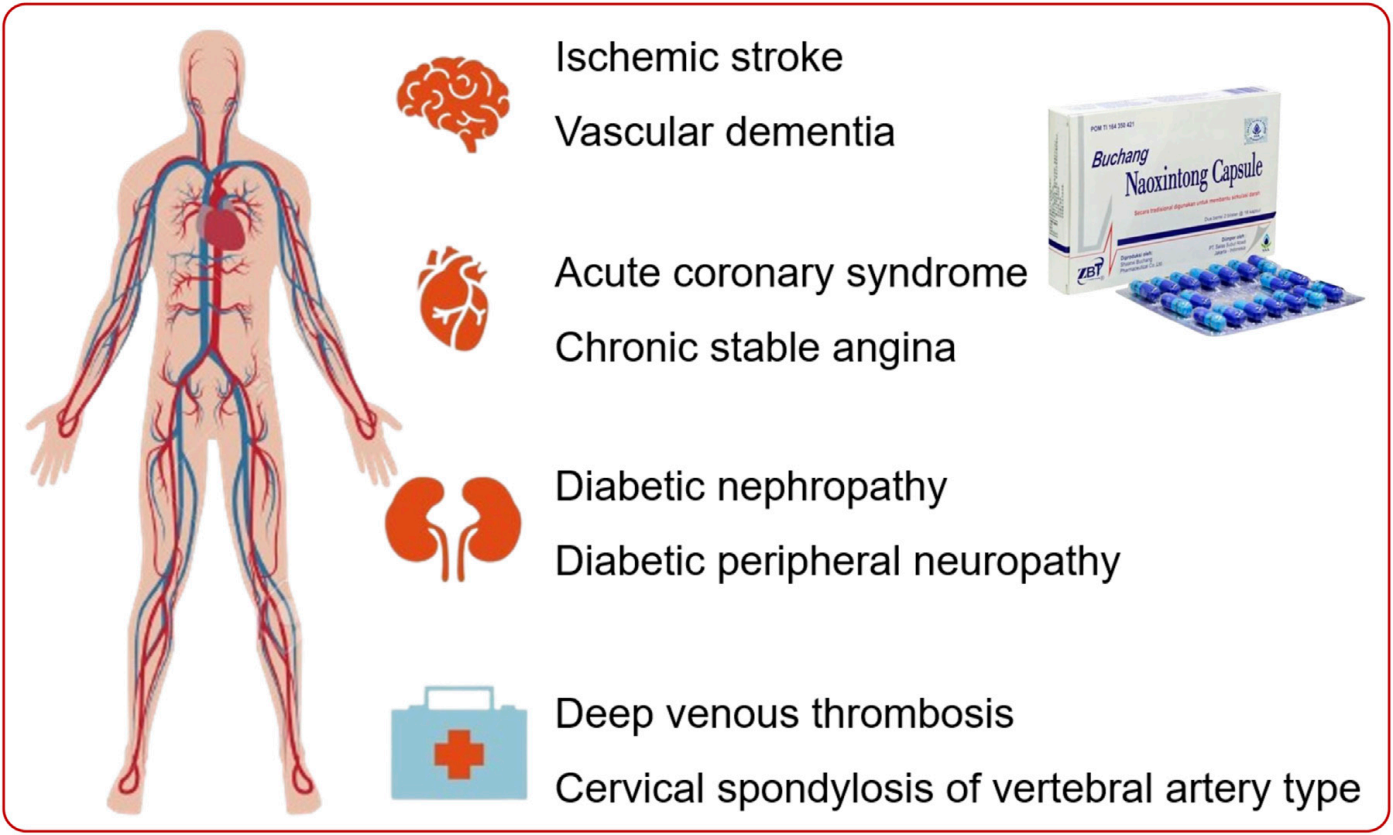
The main clinical applications of NXT.

Benefiting from the effect of promoting blood circulation and dredging collaterals, NXT is also engaged in the treatment of other diseases. Compare with rivaroxaban alone, NXT combination was more effective in improving pulmonary function index, reducing IL-6, CRP and TNF-α levels, and improving the coagulation function in patients with acute pulmonary embolism (Sun et al., 2019). The significant superiority of NXT combination was also documented in treating lower extremity deep venous thrombosis, vertebral artery cervical spondylopathy and lumbar disc herniation (Li et al., 2018). The clinical applications of NXT were outlined in Figure 3.

## 7 Toxicity and adverse reaction

A long-term preclinical toxicity test observed no adverse reactions in body weight, blood routine, heart, liver and kidney functions, and electrocardiogram (Long-Tao and Chang-Geng, 2018). In clinical application, some adverse events were reported, mainly including mild gastrointestinal reaction, such as anorexia, nausea, and diarrhea (Hou et al., 2014). Very few patients appeared skin pruritus, peeling, papules, lethargy, upset, and dizziness after starting the drug, which will disappear soon after it is withdrawn. On the whole, NXT possesses a very high safety profile in not only animal studies but also clinic trials. Further large-scale in-depth clinical safety evaluation based on big data would be meaningful to confirm the safety of NXT.

## 8 Conclusions and future remarks

Since its inception in the 1993s, NXT has been employed in a mass of researches concerning its post-marketing quality and efficacy, including chemical profiles, quality control, pharmacokinetics, pharmacological properties and clinical applications. This review outlines the existing research findings, points out some typical problems and offers suggestions to contribute to the progress of NXT.

Firstly, the quality control of NXT needs further improvement. Currently, the chemical profiles of NXT have been systematically investigated. A variety of constituents, such as amygdalin, paeoniflorin, salvianolic acid B, ligustilide, gallic acid, hydroxysafflor yellow A, and butylidenephthalide were identified as the major cardioprotective components contained in NXT (Han et al., 2019). And, several quantitative methods for simultaneous determination of multiple components in NXT have been established. However, the existing quality control methods as well as chromatographic fingerprints are still not capable of detecting the components derived from animal medicinal materials in NXT, which are insufficient to reflect the integrity of the formula. For monitoring the overall quality, it is better to further establish the chromatographic fingerprints containing the animal medicinal ingredients, which remains a big challenge. Besides, the effective substances of NXT are not yet clear. It is urgently need to clarify the contribution of the component to the pharmacodynamic effects of NXT, especially those belong to the Zoological drugs. And, the biopotency-associated multi-index content determination method should be developed. In addition, in consideration of safety, it is necessary to monitor the content of amygdalin derived from Persicae Semen and buthatoxin derived from Scorpio in NXT.

Secondly, pharmacokinetic properties of NXT still need further comprehensive study. Indeed, there are a large number of absorbable and poorly absorbed components in NXT, thereby the pharmacokinetic behavior of NXT is extremely complex. The absorption and metabolism of NXT *in vivo* simply represented by the pharmacokinetic curves of a few or dozens of components is insufficient to fully understand its pharmacokinetic behavior, which still needs further study. Besides, pharmacokinetic process of NXT in humans have not yet been fully elucidated. And, the correlation between pharmacokinetics and pharmacodynamics of NXT remains largely obscured.

Thirdly, in-depth mechanism studies are scarce. Gut-organ axis is a topic gotten more attention in research field of human health in recent years, including the gut-brain axis, gut-heart axis, gut-liver axis and so on. Since NXT is taken orally, it inevitably interacts with gut microbiota. The recent studies addressing the influences of gut microbiota offered new perspectives on the mechanism of NXT in the prevention and treatment of diseases. However, these existing studies just investigated the correlation between gut microbiota and clinical/metabolomic parameters after the NXT intervention and what the result offers is a possibility. The correlations do not prove cause and effect. Further studies should use the germfree animal techniques and fecal microbiota transplantation to confirm the causal effect of NXT on gut microbiota. Besides, further studies could also focus on the specific metabolites via targeted metabolomics technology. Specially, BAs and SCFAs have been reported to be closely associated with glucose and lipid metabolism. It would be valuable to conduct targeted studies on bile acid metabolic pathways or SCFA metabolic pathways since NXT was reported to have effect on these metabolites. And, it is encouraged to clarify functional mechanisms of NXT in human, integrating multi-omics technologies (gut microbiome, metabolomics, etc.), accompanying by verification through the animal or cell experiments.

Fourthly, high-quality RCTs are still lacking. Large-scale, multicenter randomized clinical studies with placebos, long follow-up and well-defined endpoints are urgent need for reconfirming the effectiveness and safety of NXT. Besides, since NXT is usually used in the combination with other drugs in the clinic, it is necessary to pay attention to the drug-drug interactions for ensuring medication safety. Further clinical studies can also focus on the pharmacokinetic and pharmacodynamic interactions between NXT and co-administered drugs, including antiplatelet drugs, statins and other TCMs.

In summary, NXT is a representative TCM formula in the treatment of CCVD, which could exert potent effect in relieving atherosclerosis, myocardial infarction injury, cerebral ischemia and I/R injury, thrombosis, and diabetic nephropathy, but there are also some problems need to be settled. Further studies should focus on the continued improvement of quality control, pharmacokinetic properties, pharmacological mechanism, and the reconfirmation of clinical effectiveness and safety.
